# Supplementary material for: Deep learning of human polyadenylation sites at nucleotide resolution reveals molecular determinants of site usage and relevance in disease
Source: Nat Commun. 2023 Nov 15;14:7378. doi: 10.1038/s41467-023-43266-3 (PMC10651852; doi:10.1038/s41467-023-43266-3)
Supplement: Supplementary file 1 — Supplementary information [file 41467_2023_43266_MOESM1_ESM.pdf]

## SUPPLEMENTARY INFORMATION

# Deep learning of human polyadenylation sites at nucleotide resolution reveals molecular determinants of site usage and relevance in disease

Emily Kunce Stroup and Zhe Ji

This PDF includes:

Supplementary Fig. 1 to 13

Supplementary Table 1 and 2

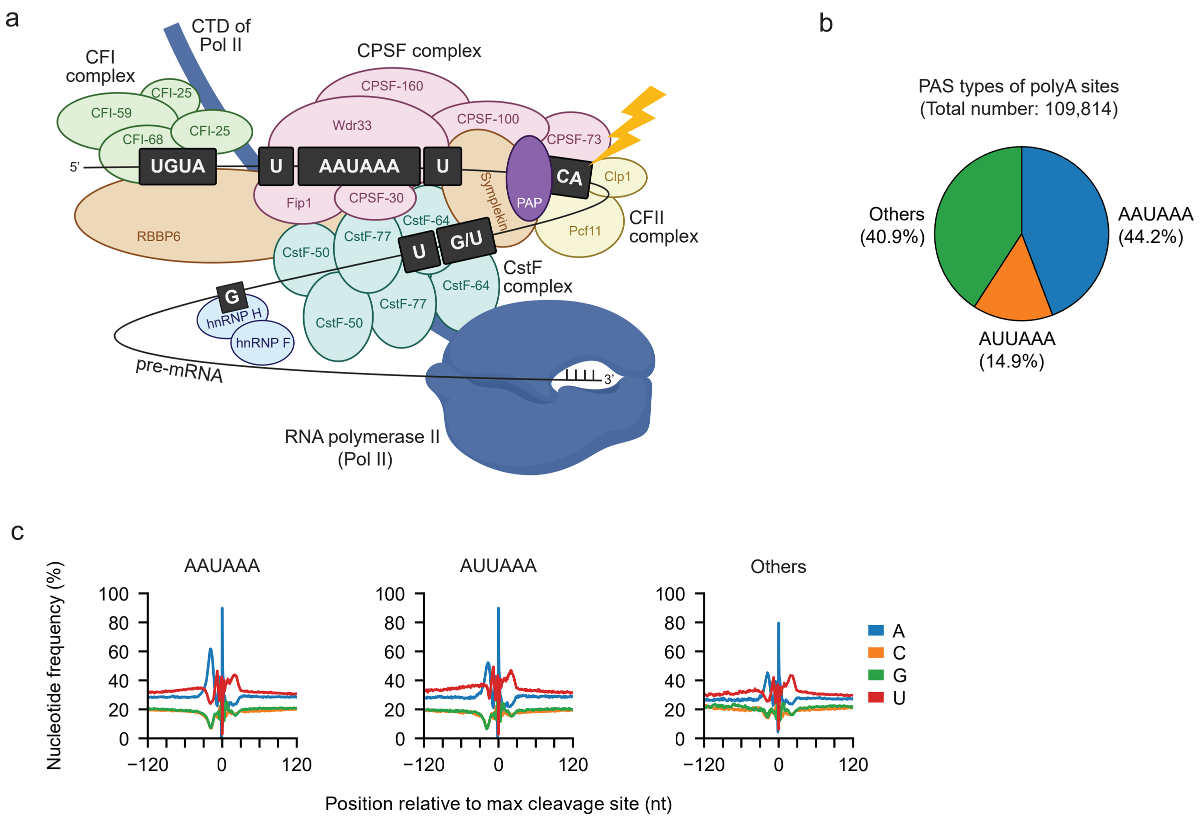


**Supplementary Figure 1. Identifying genome-wide polyA sites using 3'READS.**
(a) Schematic diagram of the polyadenylation machinery.
(b) The distribution of different PAS types.
(c) Nucleotide profiles surrounding the polyA cleavage sites, grouped by PAS type.

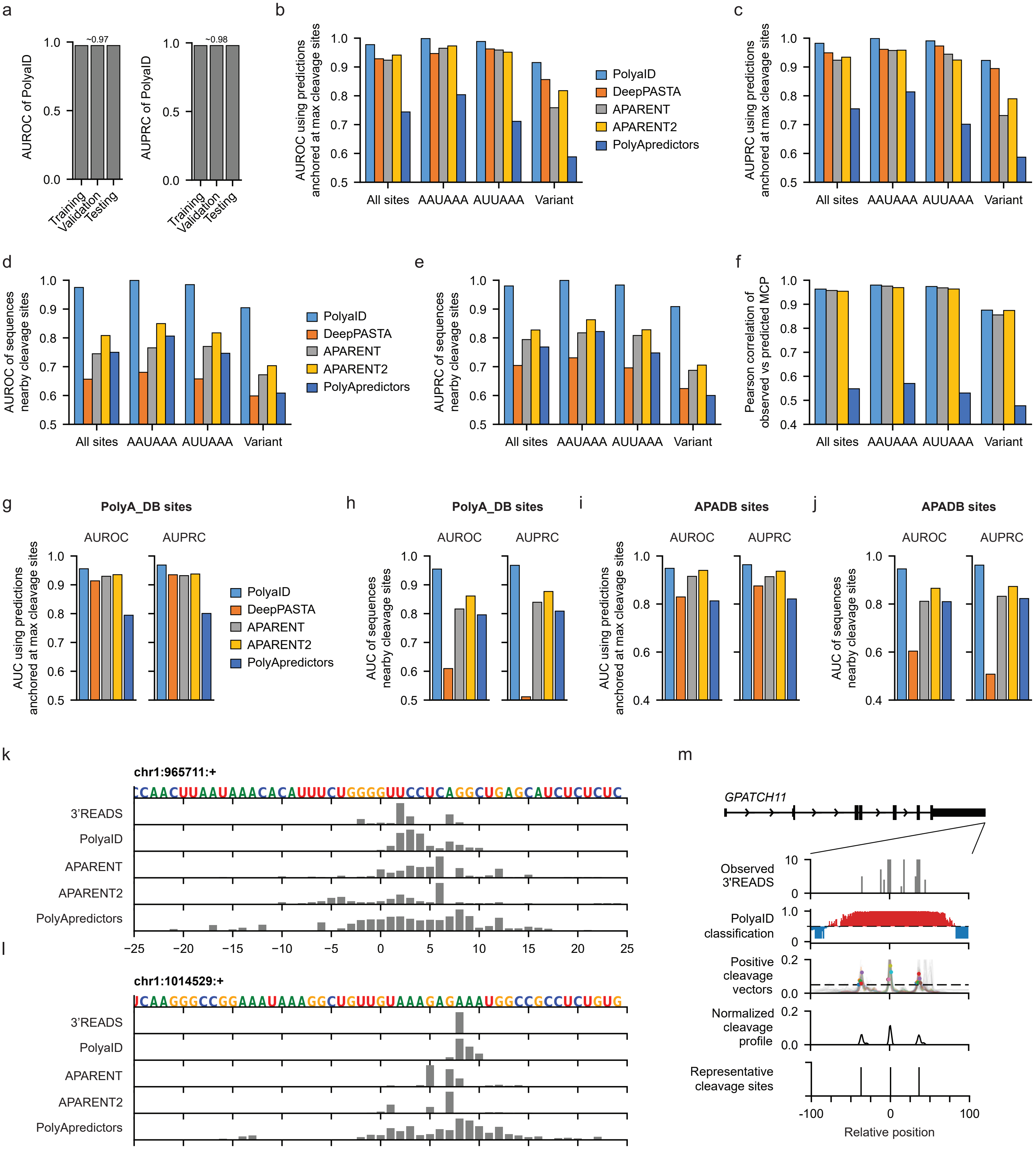

a
AUROC of PolyaID
AUPRC of PolyaID
~0.97
~0.98
Training
Validation
Testing
b
AUROC using predictions anchored at max cleavage sites
c
AUPRC using predictions anchored at max cleavage sites
All sites
AAUAAA
AUUAAA
Variant
PolyaID
DeepPASTA
APARENT
APARENT2
PolyApredictors
d
AUROC of sequences nearby cleavage sites
e
AUPRC of sequences nearby cleavage sites
f
Pearson correlation of observed vs predicted MCP
g
PolyA_DB sites
AUC using predictions anchored at max cleavage sites
h
PolyA_DB sites
AUC of sequences nearby cleavage sites
i
APADB sites
AUC using predictions anchored at max cleavage sites
j
APADB sites
AUC of sequences nearby cleavage sites
AUROC
AUPRC
k
chr1:965711:+
3'READS
PolyaID
APARENT
APARENT2
PolyApredictors
l
chr1:1014529:+
m
GPATCH11
Observed 3'READS
PolyaID classification
Positive cleavage vectors
Normalized cleavage profile
Representative cleavage sites
Relative position

**Supplementary Figure 2. Evaluating the algorithm performance on polyA site identification and cleavage predictions.**
(a) The AUROC (left) and AUPRC (right) values measuring the PolyaID performance on the training, testing, and validation datasets.
(b) The AUROC values comparing the algorithms classifying polyA sequences vs. random nucleotides. The positive sequences for the predictions were those surrounding the maximum cleavage sites from the holdout test set. The same evaluation dataset was used to compare the performance of the algorithms.
(c) Similar to (b), except showing the AUPRC values using the same dataset for evaluation.
(d) Similar to (b), except that for the positive polyA sequences, we allowed random shifting from -25 to +25 nt around the maximum cleavage sites.
(e) Similar to (d), except showing the AUPRC values.
(f) The Pearson correlation coefficient of observed vs. predicted mean cleavage position (MCP) of the polyA sites.
(g-j) The AUROC and AUPRC values comparing the models classifying sequences surrounding polyA sites defined by published databases, Polya_DB and APADB. For (g,i), we performed the PolyaID predictions using the sequences centered at the cleavage sites. For (h,j), we used the sequences randomly shifting -25nt to +25 nt from the cleavage sites for the prediction.
(k-l) Example polyA sites showing the cleavage profiles predicted by PolyaID, APARENT, APARENT2, and PolyApredictors. The Y-axis values were normalized to the maximum in the predicted vector. The polyA sites were annotated as chromosome:position:strand.
(m) The analysis steps to identify representative polyA sites within the 3'UTR of the example gene GPATCH11, which has multiple sites located in close proximity. We performed a PolyaID prediction using the 240-nt nascent RNA sequence as the input with 1-nt per step. The example results between -100 to +100 nt around the maximumly expressed cleavage site were shown.

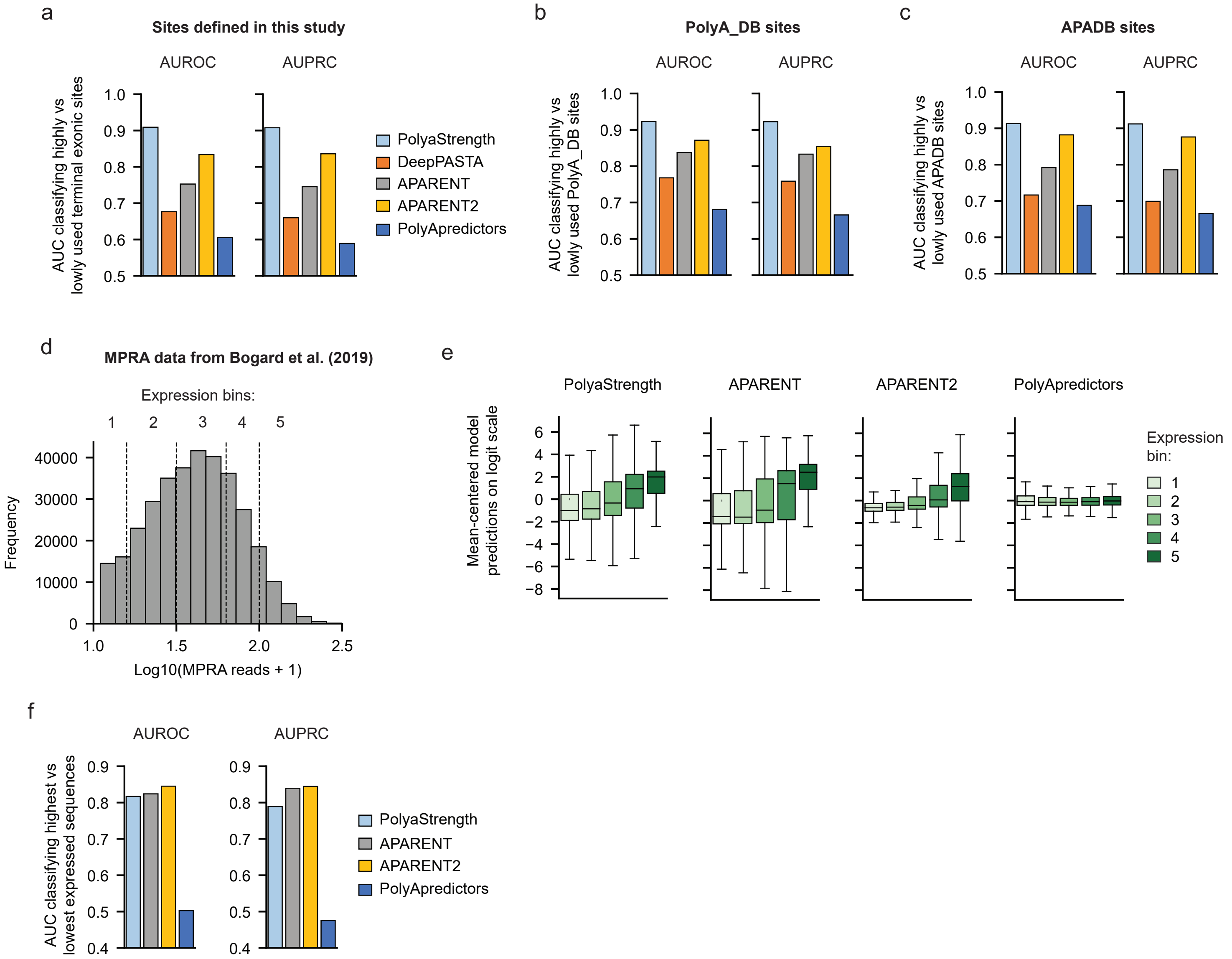


**Supplementary Figure 3. Evaluating the algorithm performance on polyA site strength discrimination.**
(a-c) The AUROC (left) and AUPRC (right) values comparing the algorithms classifying highly vs. lowly used terminal exonic polyA sites defined in this study (a), PolyA_DB (b), and APADB (c).
(d) The distribution of supporting reads for sequences from the MPRA data. We binned sequences into 5 groups based on their expression levels.
(e) The distribution of the PolyaStrength, APARENT, APARENT2, and PolyApredictors scores for the groups of sequences from the MPRA data.
(h) The AUROC (left) and AUPRC (right) values for classifying the highly vs. lowly expressed sites (group 5 vs. group 1) from the MPRA data.

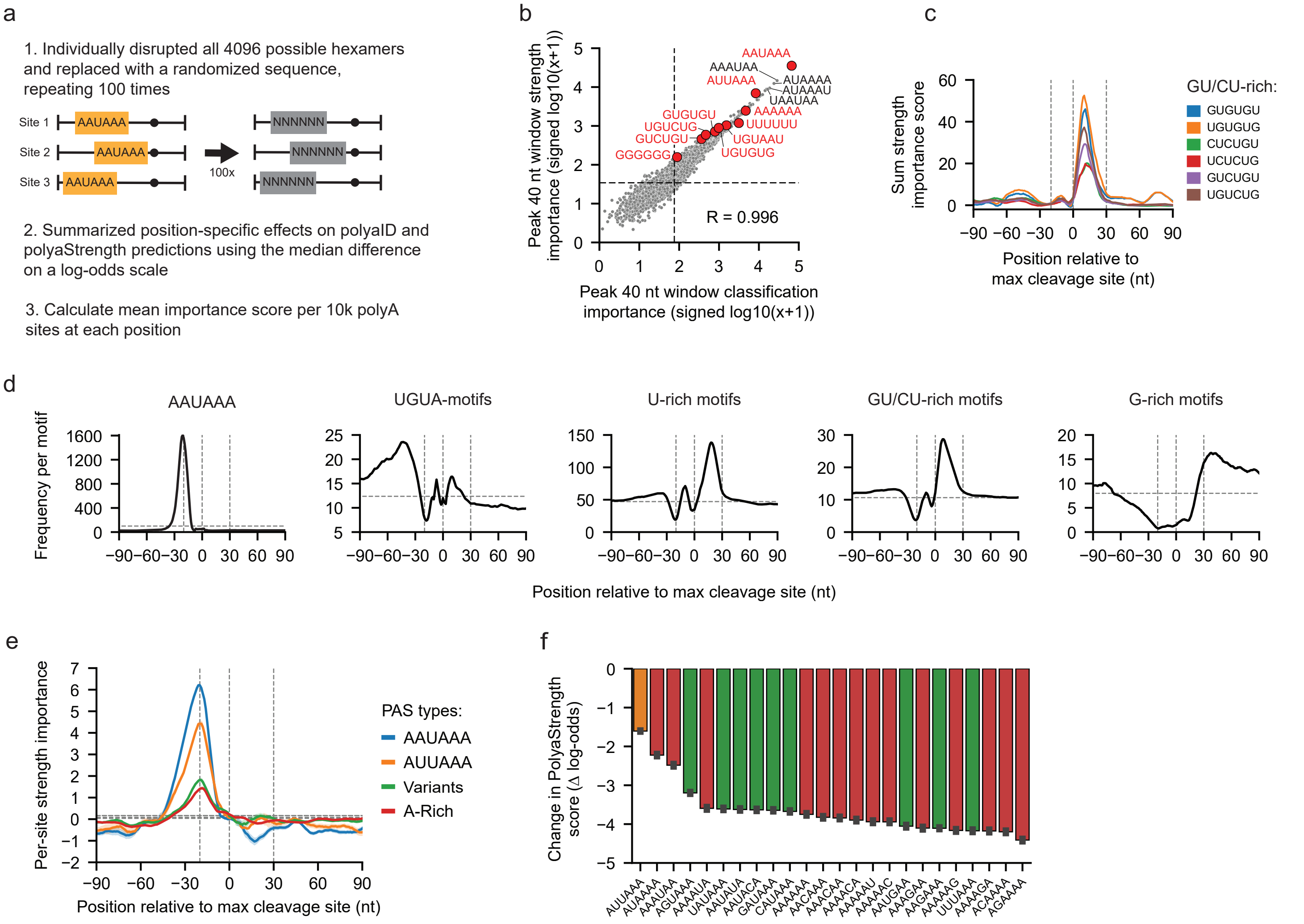


**Supplementary Figure 4. Analysis of cis-regulatory elements mediating polyadenylation activity.**
(a) Schematic diagrams showing our analysis steps to quantify position-specific motif importance scores using the PolyaID and PolyaStrength models.
(b) Correlation between motif importance scores calculated by the PolyaID and PolyaStrength models. Importance scores have been transformed using the signed log10(importance + 1). The Pearson correlation coefficient value is shown. The dotted lines represent the 99.99th percentile of expected motif importance scores.
(c) The distribution of sum importance scores of individual GU/CU-rich hexamers around polyA sites.
(d) The frequency of indicated hexamer types around polyA sites.
(e) The distribution of summed importance scores of PAS hexamers around polyA sites. The lighter, shaded regions show the standard error of the summed importance score.
(f) We replaced AAUAAA with other PAS variants and then calculated the change in PolyaStrength scores (logit). When we performed the replacement to non-AUUAAA variants, we required that no new AAUAAA/AUUAAA would be created. The mean and 95% confidence intervals are shown (N = 23,793 AAUAAA signals analyzed).

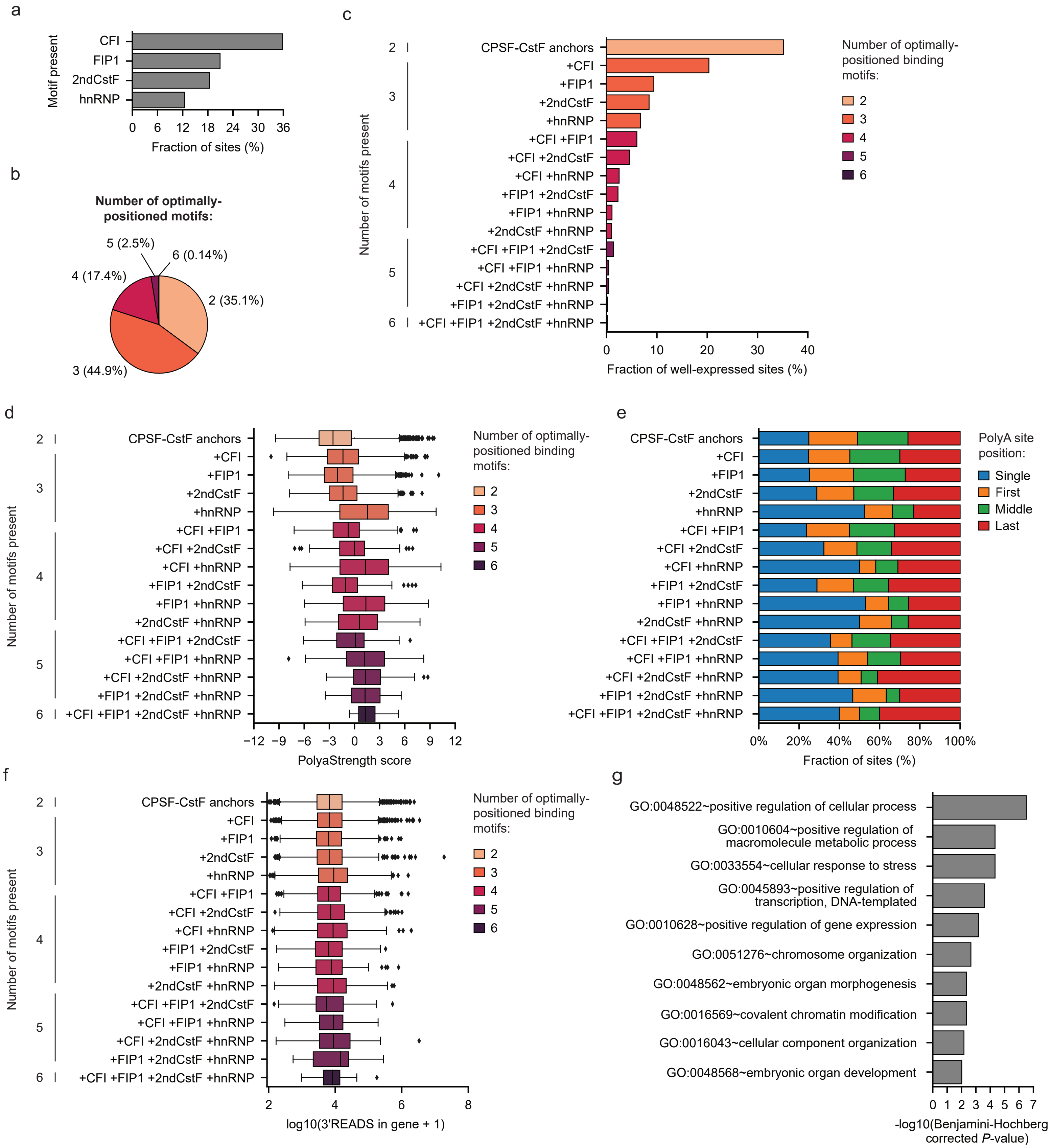


**Supplementary Figure 5. Analyzing motif compositions of genomic polyA sites.**
(a) For the 13,876 polyA sites with the PAS AAUAAA and at least one downstream U-rich or GU/CU-rich motif, we examined the fractions containing another binding motif for the indicated factors.
(b) The fraction of sites with 2-6 optimally-positioned motifs.
(c) The fraction of sites containing other indicated motifs or motif combinations.
(d) The distribution of PolyaStrength Scores.
(e) The distribution of polyA site types.
(f) The distribution of gene expression levels calculated as the sum of 3’READS in each gene.
(g) Gene ontology showing enriched biological process terms for genes expressing pA sites with ≥5 optimally-positioned motifs.

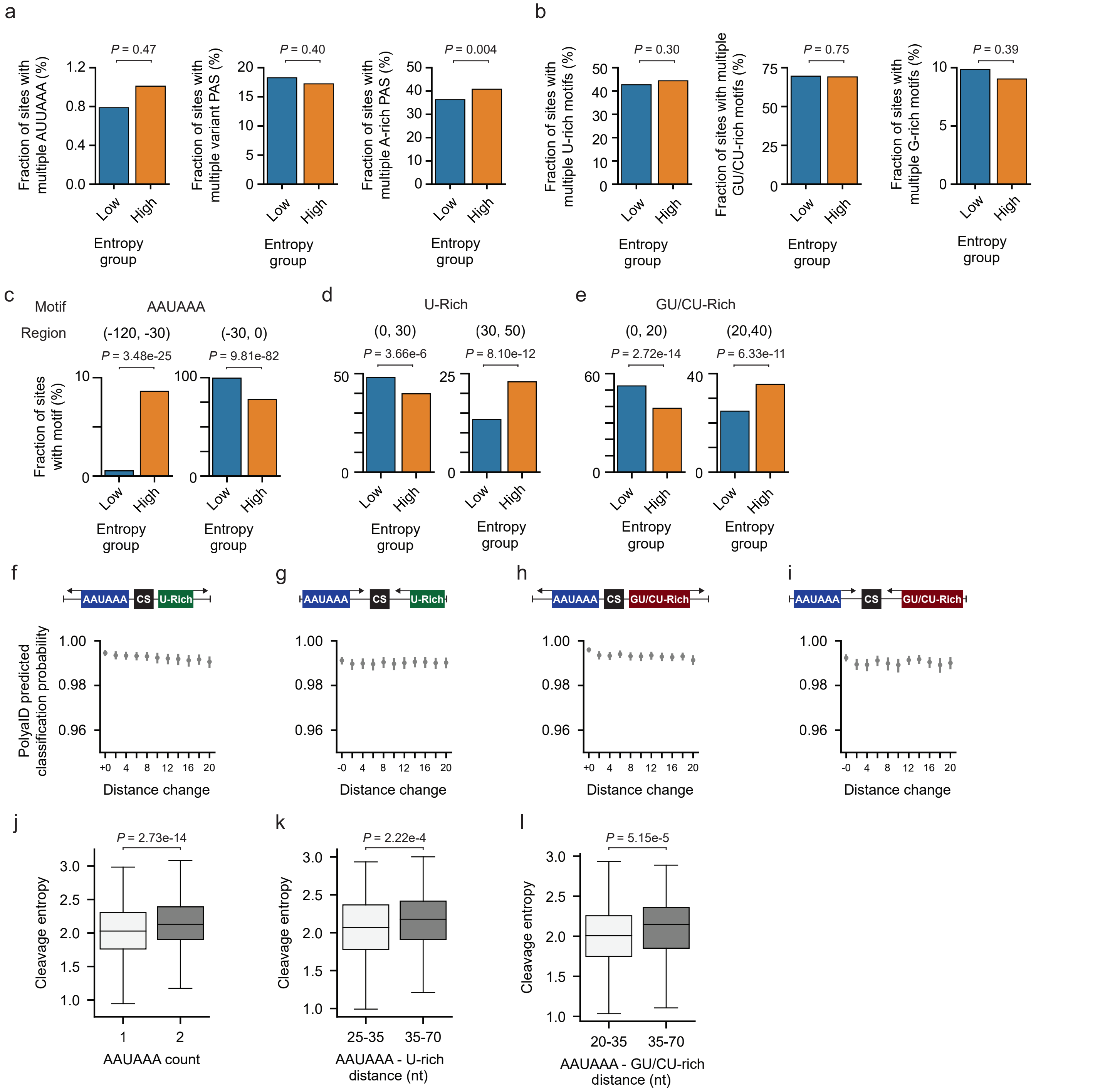


**Supplementary Figure 6. Comparing the polyA site groups with high vs. low cleavage site heterogeneity.**
(a) The fractions of polyA sites using the indicated PAS types in the two groups. The P-values of two-sided two proportion tests are shown.
(b) The fractions of polyA sites with multiple downstream U-rich, GU/CU-rich, and G-rich elements. The P-values of two-sided proportion tests are shown.
(c-e) The fraction of polyA sites containing the cis-elements in the indicated regionsThe P-values of two-sided proportion tests are shown.
(f-i) We moved the AAUAAA and U-rich (or GU/CU-rich) elements farther apart or closer together (1 nt each per step), and then examined PolyaID-predicted classification probability. The mean and 95% confidence intervals are shown. N = 676, 635, 949, and 658 sites used for f-i, respectively.
(j) The distribution of observed cleavage entropy for sequences from the MPRA data that contained either 1 or 2 AAUAAA signals. We randomly selected an equal number of sites with 1 AAUAAA to match the number with 2 AAUAAA (N = 1493 in each group). The Wilcoxon rank sum test P-value is shown.
(k) The distribution of observed cleavage entropy for sequences from the MPRA data, grouped based on the AAUAAA and downstream U-rich distances (i.e. 25-35 nt vs. 35-70 nt; N = 477 and 383, respectively). The Wilcoxon rank sum test P-value is shown.
(l) Similar to (k), except grouped based on the distances between AAUAAA and downstream GU/CU-rich elements (i.e. 20-35 nt vs. 35-70 nt; N = 923 and 222, respectively). The Wilcoxon rank sum test P-value is shown.

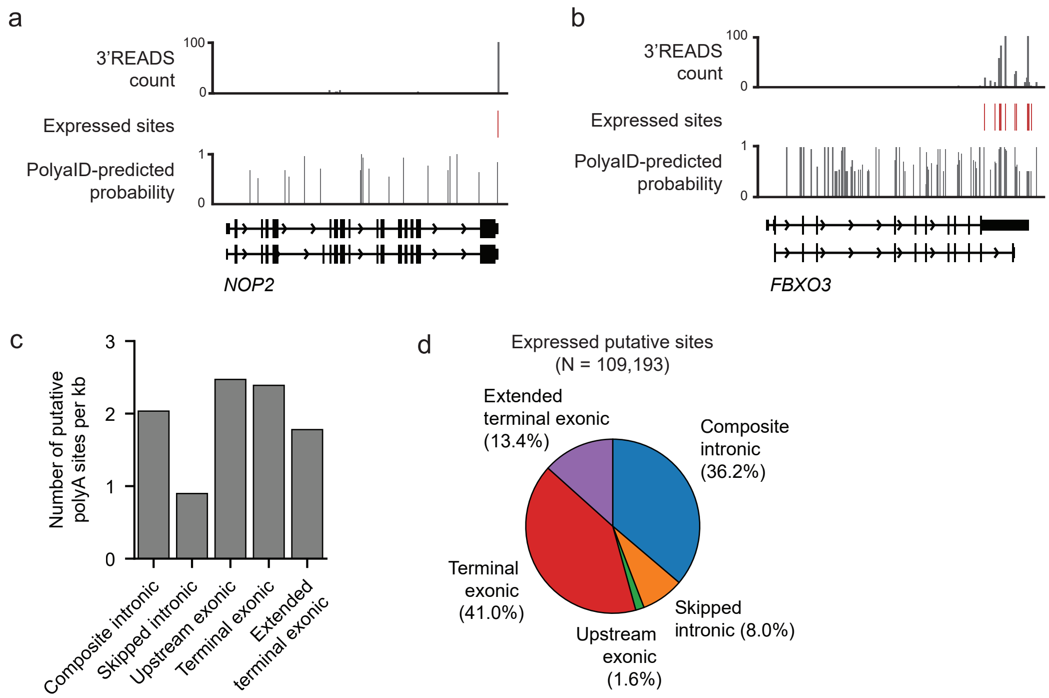


**Supplementary Figure 7. Differential usage of PolyaID-predicted putative polyA sites.**
(a-b) Example genes showing the location of PolyaID-predicted putative sites (classification probability >0.5), 3'READS distribution, and the expressed sites supported with ≥10 PASS reads.
(c) Number of putative polyA sites per kb in different genomic regions.
(d) The distribution of expressed polyA sites grouped based on genomic location.

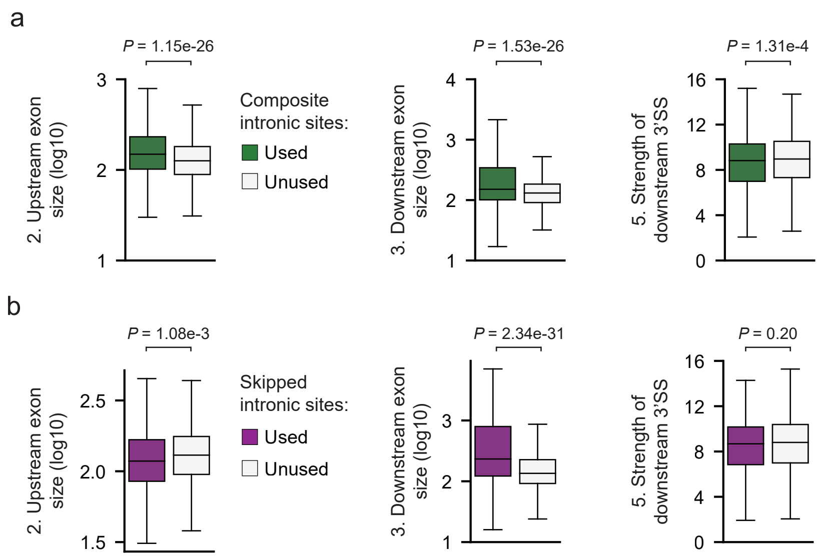


**Supplementary Figure 8. Examining genomic features regulating intronic polyA site expression.**
(a) We controlled for the polyA site strength and then compared selected genomic features between used (green) and unused (gray) composite intronic sites. The Wilcoxon rank sum test *P*-values were shown.
(b) We controlled for the polyA site strength and then compared selected genomic features between used (magenta) and unused (gray) skipped intronic sites. The Wilcoxon rank sum test *P*-values were shown.

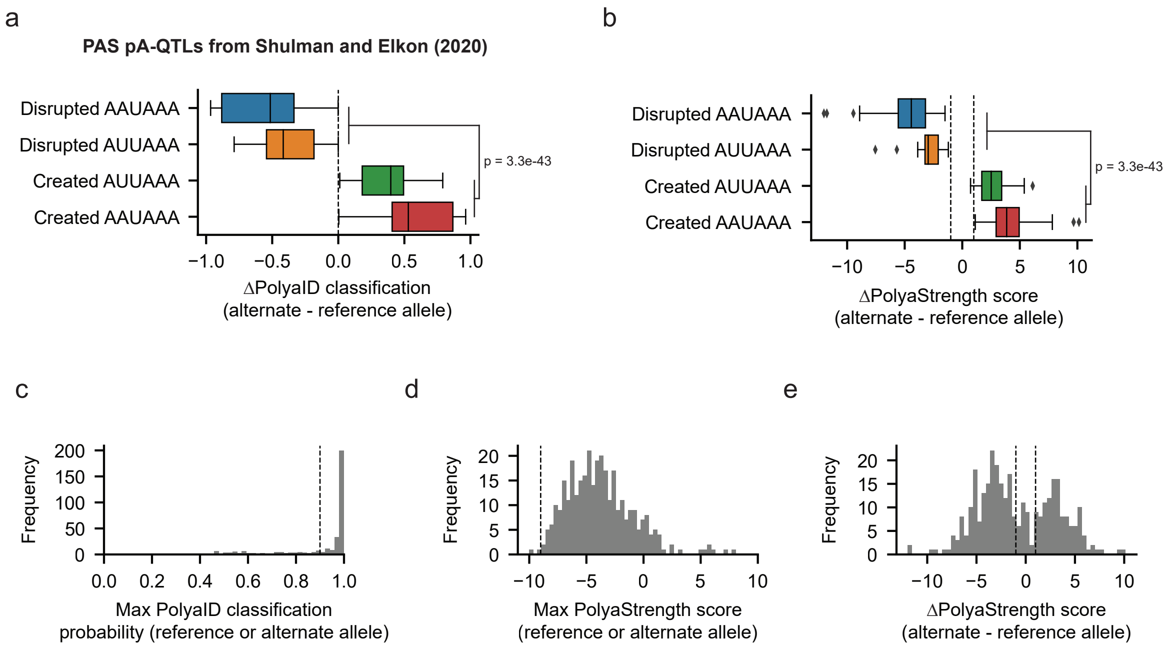


**Supplementary Figure 9. The analyses of PAS pA-QTL variants.**
(a-b) ΔPolyaID classification (a) and ΔPolyaStrength scores (b) comparing the alternate–reference allele predictions for PAS pA-QTLs. PAS pA-QTLs were split by their effect on AAUAAA or AUUAAA polyadenylation signals. The Wilcoxon rank sum test *P*-value comparing PAS creation vs. disruption was shown (N = 105 and 161, respectively).
(c-e) The distribution of PolyaID classification probabilities (c), PolyaStrength scores (d), and ΔPolyaStrength scores (e) for the selected PAS pA-QTLs. The cutoffs empirically determined from this analysis and used to identify significant variants in down-stream genetics analyses were indicated as dashed lines.

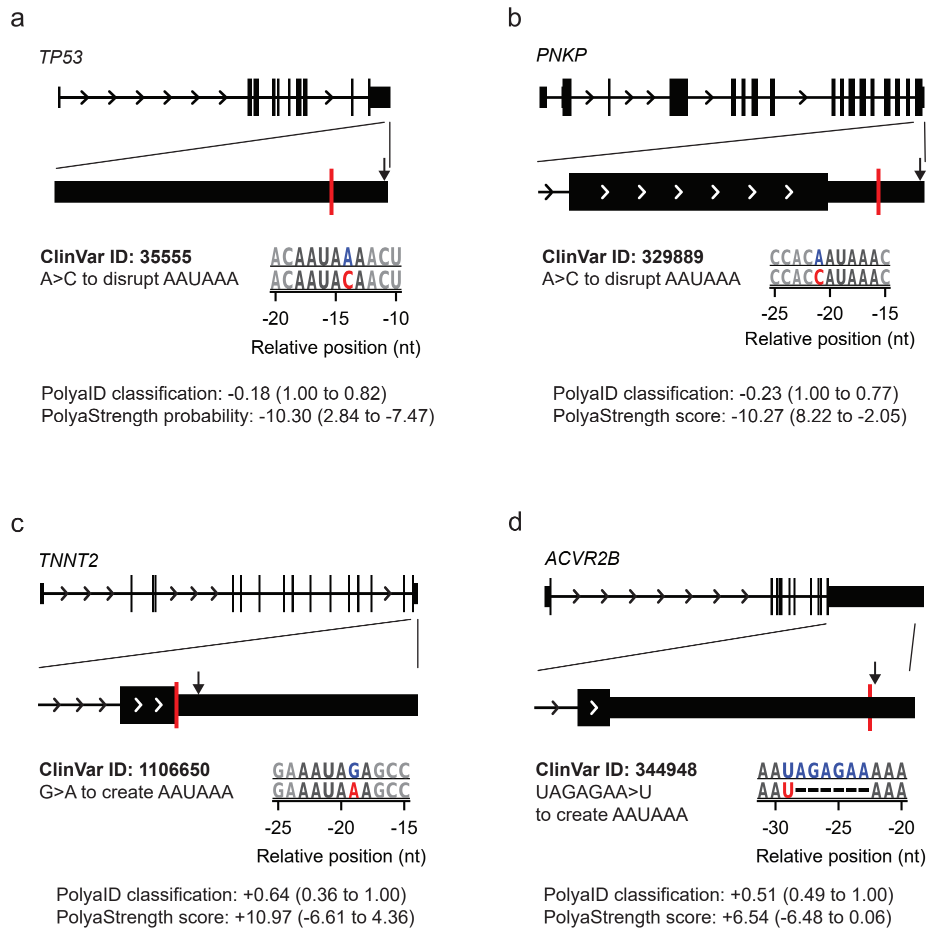


**Supplementary Figure 10. Identifying disease/trait-associated variants regulating polyadenylation activity using our models.**
(a-d) Example variants altering polyadenylation activity. For each variant, we show the variant ID, affected motif type, the PolyaID classification score changes, and the PolyaStrength score changes.

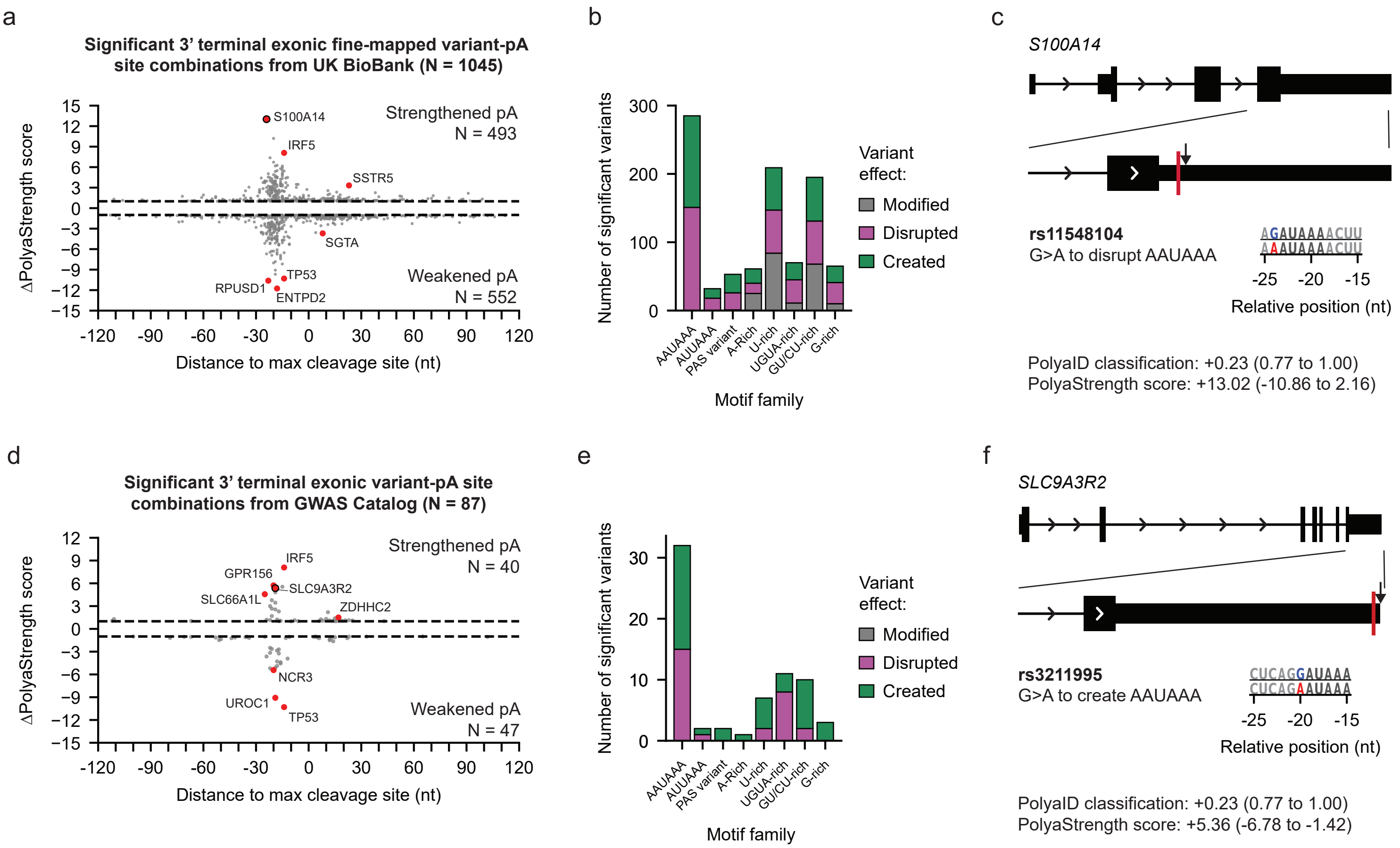


**Supplementary Figure 11. Identifying variants located in 3'-terminal exons regulating polyadenylation activity from GWAS Catalog and UK BioBank.**

(a) Effects of significant fine-mapped variants from the UK BioBank on the PolyaStrength score versus the distance to the maximum cleavage site (N = 1045 variant-pA site combinations). Dashed lines indicate the ΔPolyaStrength cutoff at +/- 1.

(b) Frequency of motif families affected by UK BioBank variants (N = 964 significant variants that modified, disrupted, or created the indicated motif family).

(c) An example variant predicted to alter polyadenylation activity in the *S100A14* gene. For each variant, we show the variant ID, affected motif type, the PolyaID classification score changes, and the PolyaStrength score changes.

(d) Similar to (a), but showing GWAS Catalog variants (N = 87 variant-pA site combinations).

(e) Similar to (b), but showing the motif families affected by GWAS Catalog variants (N = 81 significant variants).

(f) Similar to (c), but showing a GWAS Catalog variant in the *SLC9A3R2* gene.

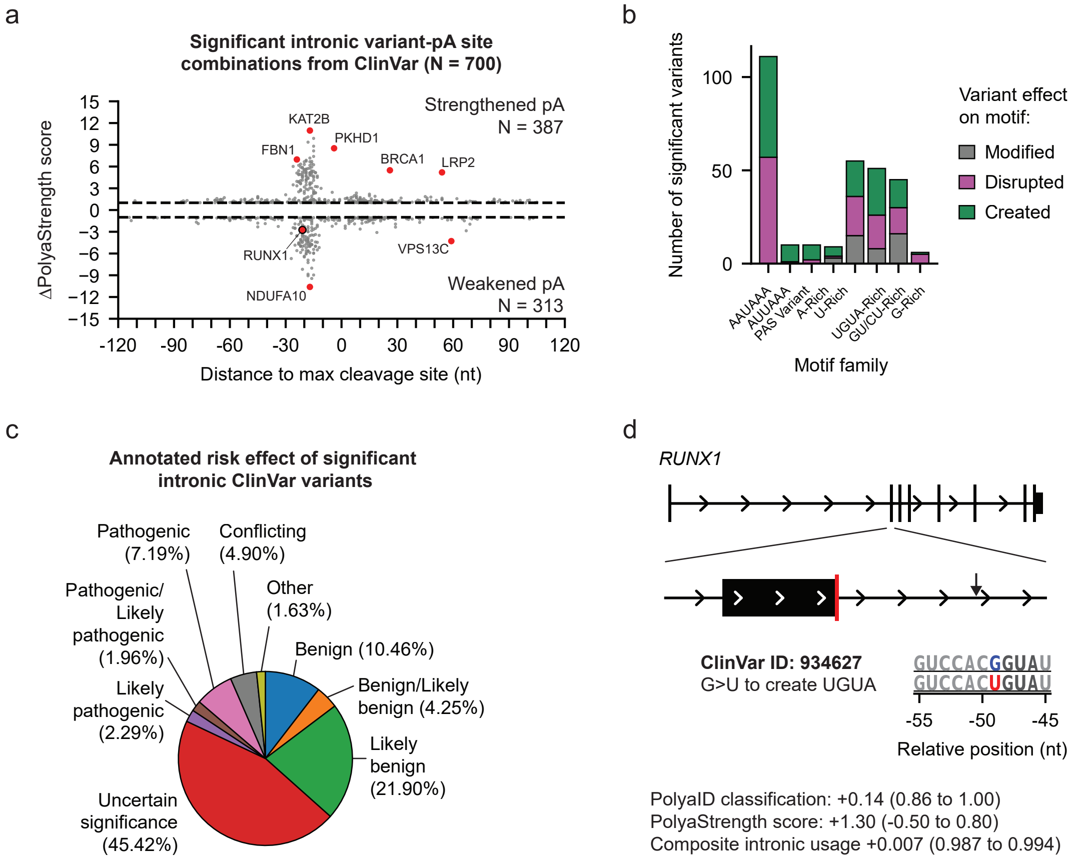


**Supplementary Figure 12. Analysis of ClinVar variants located in introns regulating polyadenylation activity.**
(a) Effects of significant intronic ClinVar variants on the PolyaStrength score versus the distance to the maximum cleavage site (N = 700 variant-pA site combinations). Dashed lines indicate the ΔPolyaStrength (logit) cutoff at +/- 1. The number of variants predicted to strengthen or weaken the associated pA site was indicated.
(b) Frequency of motif families affected by intronic ClinVar variants (N = 297 significant variants that modified, disrupted, or created the indicated motif family).
(c) The annotated risk effects of significant intronic ClinVar variants altering polyadenylation activity (N = 306 significant variants).
(d) Example intronic variant altering polyadenylation activity in the *RUNX1* gene. We present the variant ID, affected motif type, the PolyaID classification score changes, the PolyaStrength score changes, and the expression probability calculated using the relevant logistic regression model shown in Fig. 5.

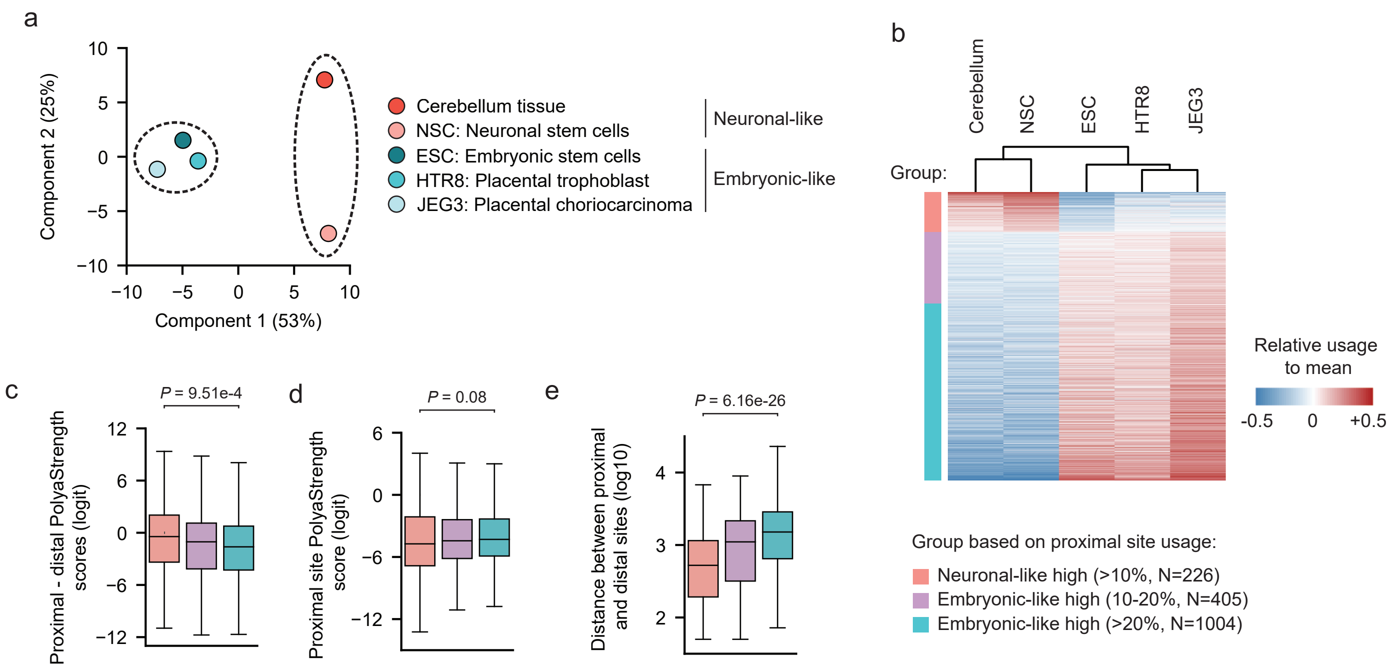


**Supplementary Figure 13. Tissue-specific polyA site usage in neural and embryonic tissues.**
(a) The principal component analyses of the samples using relative usage levels of proximal polyA sites showing APA regulation across five tissue/cell types (see Methods for details of APA analyses). The percentages of variance explained by the PC1 and PC2 were shown in parentheses.
(b) The heatmap showing the mean-centered relative usage levels of proximal polyA sites across the five tissue cell types. We grouped the proximal sites into those showing higher expression in neuronal-like cells (cerebellum and NSCs), and these with higher expression in embryonic-like cells (ESCs, HTR8 and JEG3) which were further divided into 2 groups (10~20% and >20% higher usage). The number of proximal polyA sites in each group was indicated in the heatmap legend.
(c) The relative difference between logit-based PolyaStrength scores (proximal - distal). The Wilcoxon rank-sum test *P*-value comparing the indicated group were shown.
(d) The absolute PolyaStrength scores of proximal polyA sites. The Wilcoxon rank-sum test *P*-value comparing the indicated group were shown.
(e) The distance between the differentially used proximal site and paired distal site by usage category. The Wilcoxon rank-sum test *P*-value comparing the indicated group were shown.

**Supplementary Table 1. The annotated features for intronic polyA sites.**

| Type | Features | Measurement |
|---|---|---|
| Composite, skipped | PolyA site strength | PolyaStrength Score (log-odds) |
| Composite, skipped | Distance to the nearest upstream 5’SS | log10(distance) |
| Composite, skipped | Upstream 5’SS strength | MaxEntScan Score |
| Composite, skipped | Downstream 3’SS strength | MaxEntScan Score |
| Composite, skipped | Size of upstream exon | log10(exon size) |
| Composite, skipped | Size of downstream exon | log10(exon size) |
| Composite, skipped | Located in the last intron? | 1: yes; 0: no |
| Composite | Ratio of intron size and distance to upstream 5’SS | log2(intron size/distance to 5’SS) |
| Skipped | Distance to the first upstream 3’SS | log10(distance) |
| Skipped | First upstream 3’SS strength | MaxEntScan Score |
| Skipped | Ratio of distance to upstream 3’SS and distance to downstream 3’SS | log2(distance to upstream 3’SS/ distance to downstream 3’SS) |

**Supplementary Table 2. The annotated features for terminal exonic APA sites.**

| Type | Features | Measurement |
|---|---|---|
| Proximal, distal | PolyA site strength | PolyaStrength Score (log-odds) |
| Proximal, distal | Relative distance between two sites | log10(distance) |
| Proximal, distal | Distance to downstream gene in the opposite strand | log10(distance) |
| Proximal, distal | Size of last intron | log10(intron size) |
