# Supplementary material for: Deep learning of human polyadenylation sites at nucleotide resolution reveals molecular determinants of site usage and relevance in disease
Source: Nat Commun. 2023 Nov 15;14:7378. doi: 10.1038/s41467-023-43266-3 (PMC10651852; doi:10.1038/s41467-023-43266-3)
Supplement: Supplementary file 3 — Description of Additional Supplementary Files [file 41467_2023_43266_MOESM3_ESM.pdf]

Description of Additional Supplementary Files

File Name: Supplementary Data 1
Description: The 3'READS datasets used in the analyses.

File Name: Supplementary Data 2
Description: The polyA sites identified using 3'READS datasets.

File Name: Supplementary Data 3
Description: Parameters for the PolyaID and PolyaStrength deep learning models.

File Name: Supplementary Data 4
Description: *Cis*-regulatory motifs regulating polyadenylation activity.

File Name: Supplementary Data 5
Description: Predictions for the PAS pA-QTLs.

File Name: Supplementary Data 6
Description: The annotation of ClinVar, UK BioBank, GWAS Catalog variants impacting polyadenylation activity.
